# Laser Welding of ARMOX 500T Steel

**DOI:** 10.3390/ma17143427

**Published:** 2024-07-11

**Authors:** Aleksander Lisiecki, Agnieszka Kurc-Lisiecka, Wojciech Pakieła, Grzegorz Chrobak, Gilmar Ferreira Batalha, Marcin Adamiak

**Affiliations:** 1 Department of Welding Engineering, Faculty of Mechanical Engineering, Silesian University of Technology, Konarskiego 18A Str., 44-100 Gliwice, Poland; 2 Institute of Applied Sciences, WSB Merito University in Poznan, Sportowa 29 Str., 41-506 Chorzow, Poland; agnieszka.kurc-lisiecka@chorzow.merito.pl; 3 Department of Engineering Materials and Biomaterials, Silesian University of Technology, Konarskiego 18A Str., 44-100 Gliwice, Poland; wojciech.pakiela@polsl.pl; 4 IPG Photonics Sp. z o.o., ul. Wyczółkowskiego 8 Str., 44-109 Gliwice, Poland; gchrobak@ipgphotonics.com; 5 Department of Mechatronics and Mechanical Systems Engineering, Polytechnic School of Engineering of the University of Sao Paulo (USP), São Paulo 05508-900, Brazil; gfbatalh@usp.br; 6 Materials Research Laboratory, Faculty of Mechanical Engineering, Silesian University of Technology, Konarskiego 18A Str., 44-100 Gliwice, Poland; marcin.adamiak@polsl.pl

**Keywords:** laser welding, armor steel, Armox 500T, microstructure, mechanical properties

## Abstract

The article describes the results of the study on laser welding of armor plates with a nominal thickness of 3.0 mm. The plates were made of Armox 500T steel characterized by a hardness of up to 540 HB, a minimum yield strength of 1250 MPa, an ultimate strength of up to 1750 MPa, and an elongation A5 minimum of 8%. The laser used for the welding tests was a solid state Yb:YAG laser. The influence of basic parameters such as laser output power, welding speed, and focal plane position on the weld geometry was determined during bead-on-plate welding tests. The optimal conditions for butt joint welding were determined, and the test joints were subjected to mechanical and impact tests, metallographic analysis, and hardness measurements. It has been shown that it is possible to laser weld Armox 500T armor plates, and at the same time it is possible to provide high quality butt joints, but this requires precise selection of welding parameters. A decrease in HAZ hardness of about 22–35% in relation to the hardness of the base material, ranging from 470 to 510 HV0.2, was found. The ultimate tensile strength of the test joints was approx. 20% lower than the Armox 500T steel. The bending tests revealed the low plasticity of the tested joints because the bending angle was just 25–35°. The results of Charpy V–notch test revealed that the impact toughness of the weld metal at −20 °C was approx. 30% lower than at room temperature.

## 1. Introduction

Armored vehicles must provide high ballistic protection, and on the other hand, they must have the lowest possible weight to ensure high mobility even in very difficult terrain and environments [1]. The basic structural material for such vehicles is steel because it is susceptible to plastic processing, joined via welding, and facilitates the shaping of functional properties in a wide range as a result of heat treatment [1,2]. Steels that provide good ballistic properties are usually ultra-high strength (UHS) steels processed by quenching and tempering (Q&T). High mechanical properties are provided by martensitic microstructure. However, due to subsequent tempering, the ductility and toughness is increased, providing better impact energy absorption which is crucial for ballistic performance. Many researchers involved in testing the ballistic characteristic of armor plates made of different grades of armor steel indicate that the higher the hardness of the steel obtained as a result of tempering, the higher the ballistic resistance [3,4,5]. Therefore, tempering takes place at a relatively low temperature, retaining high hardness and thus high ballistic performance [1,6].

The most commonly used joining methods of the armor steel components are gas metal arc welding (GMA) and submerged arc welding (SAW). Recent research has also been conducted on hybrid laser arc welding (HLAW) [1,7,8,9,10]. Fei et al. [1] presented a novel technique of TIG welding of armor steel with a trapezoidal AISI309 austenitic stainless steel interlayer. Thanks to controlling the dilution and sequence of solidification, they provided the desired chemical composition and microstructure of the weld metal, contributing improvements in strength and toughness, as well as limiting the tendency toward both solidification cracking and hydrogen-assisted cold cracking.

Turichin et al. [7] investigated laser arc hybrid welding of ultra-high strength steels, including Armox 600T steel. They used three filler wires of different chemical compositions and determined the influence of the filler wires’ chemical composition on the microstructure and mechanical properties of the welds. The main conclusion drawn from the investigations was that the welds of 7.0 mm thick Armox 600T plates were about 14% weaker than the base metal.

Kumar et al. [8] conducted a study on shielded metal arc welding of ultra-high hard armor steel of CE equivalent 0.91 using the following different electrodes: austenitic stainless steel type, super duplex stainless steel type, and low-hydrogen ferritic type. They found that the joints made via the austenitic stainless steel electrodes showed the best impact toughness, while the joints made via low-hydrogen ferritic electrodes exhibited the best tensile strength and the highest hardness.

Skowrońska et al. [9] also investigated a hybrid process of armor steel welding. They investigated PTA-MAG (plasma transferred arc—metal active gas) welding of RAMOR 500 steel plates 6.7 mm thick with a filler type Mn4Ni2CrMo. They pointed some advantages of the welding process, including limited decreasing of mechanical properties of HAZ at an acceptable level from the point of view of maintaining the antiballistic properties of the base material.

All of the mentioned methods usually utilize austenitic type wire as the additional material because the weldability of the armor grade steel is limited due its high carbon equivalent, high hardness, and thus tendency for cold cracking. However, the use of a plastic austenitic deposit leads to a significant reduction in the ballistic performance of welded joints, as reported by Fei et al. [1] and Magudeeswaran et al. [6]. Moreover, even the plastic and relatively soft austenitic deposit does not completely solve the problem of hydrogen-induced cracking (HIC) in the heat-affected zone (HAZ) of the welded joints of armor steel [6].

A method that has not yet been used industrially for the welding of armor steel, but provides some benefits and gives the possibility of further optimization and development of welding technology of this grade of steel is laser welding [11,12,13,14,15,16,17]. Laser welding may be conducted as an autogenous process, without additional material, and thanks to efficient shielding of inert gas, the process can be considered as a low-hydrogen process. Moreover, the characteristic feature of laser welding is a narrow HAZ and a relatively low level of welding stresses, which in turn is beneficial to the reduction in the tendency for hydrogen-induced cracking. It is also worth noting that currently available lasers with a power of several dozen kW allow for welding joints with a thickness over 30 mm in one pass. The studies carried out so far in the field of laser welding of armor steel indicate that despite the low heat input of laser welding and rapid cooling, softening of HAZ occurs, which may be additionally beneficial in terms of the reduction in the tendency for hydrogen-induced cracking. Janicki [11] investigated the autogenous laser welding of butt joints of 3.6 mm thick Armox 500T steel, and within the investigated technological conditions of welding he reported a significant drop of hardness in the HAZ, up to 40% comparing to the base material. Similar results were reported by Bassett [12] who found 30% hardness drop in HAZ of laser welded 13.0 mm thick armor steel CP50.

Laser welding also shows some limitations. Since the laser beam is very a precise heat source, usually focused to a diameter of 0.2–0.3 mm, it requires high tolerance of edges preparation and positioning of the laser beam along the weld trajectory, in the case of butt joint welding. Moreover, in the case of autogenous welding, it is not possible to control the chemical composition of the weld metal, and weld undercuts are typical. These limitations make the application of laser welding on an industrial scale difficult in certain areas [15,16,17,18,19,20,21,22,23,24].

However, there are few publications in the world on the laser welding of armor steel. Therefore, research was undertaken on the technology of autogenous laser welding of armor plates in order to determine the influence of basic welding parameters on the microstructure and mechanical properties, in particular the plasticity and impact toughness of test joints also at low temperature. As a result, laser welding conditions in which weld porosity has been eliminated were determined. The obtained narrow welds, compared to arc and hybrid welding methods, may be beneficial for ensuring better ballistic performance of the entire butt joints of armor plates. Therefore, it is planned to continue research in this area, expanding the range of plates thicknesses and grades of armor steels.

Moreover, the preliminary tests of welding these armor plates with a single-mode beam and also via a unique technique with two combined laser beams, AMB (adjustable mode beam) with a total power of 6.0 kW, and beam oscillation have been already carried out. Results have shown that it is possible to further optimize the laser welding process, which ensures a favorable shape of the weld and favorable joint properties, even in the case of larger sheet thicknesses.

## 2. Materials and Methods

The armor plates with the dimensions of 250 × 250 mm used in this study were made of armor steel Armox 500T (SSAB, Stockholm, Sweden) in quenched and tempered condition (Q&T). The smallest thickness plate available from the manufacturer, intended for ballistic protection of light vehicles, was selected for the tests. The nominal thickness of the plates was 3.0 mm. Such an armor plate should provide protection against normal 7.62 mm caliber bullets (excluding armor-piercing bullets, e.g., with a tungsten core). Chemical composition and mechanical properties of Armox 500T steel, provided by manufacturer SSAB and determined within the study, are listed in Table 1 and Table 2, respectively.

The laser welding tests were conducted on a fully automated stand with the solid state Yb:YAG Disk laser (Trumpf, Ditzingen, Germany) emitted in continuous wave (cw) mode at 1.03 μm wavelength with maximum output power of 3.3 kW, and beam quality (beam parameter product—BPP) of 8.0 mm·mrad. The laser beam was focused to a diameter of 200 μm.

In the initial stage of the study, the bead-on-plate welds were produced to simulate the process of autogenous butt joint welding and to investigate the influence of the basic welding parameters on the penetration depth, weld shape, and quality of welds (e.g., presence of voids, lack of penetration, undercuts, etc.). The initial tests were conducted in the laser output power range from 1.0 to 3.0 kW, welding speed from 0.5 to 2.5 m/min, and different focal position. The detailed bead-on-plate laser welding conditions are summarized in Table 3.

Based on the initial tests, parameters providing full penetration and required weld quality (proper shape and no voids in weld) were chosen for autogenous butt joint laser welding. In order to determine the influence of energy input on the microstructure and mechanical properties of test joints, the extreme parameters with the highest (180 J/mm) and lowest (70 J/mm) energy input were chosen for laser welding of butt joints.

Specimens for welding tests were cut from the armor plate into pieces in dimensions of 120.0 × 45.0 mm via mechanical cutting. Prior to the welding test, the specimens were sandblasted and additionally the edges to be welded were cleaned with acetone.

**Figure 1:**
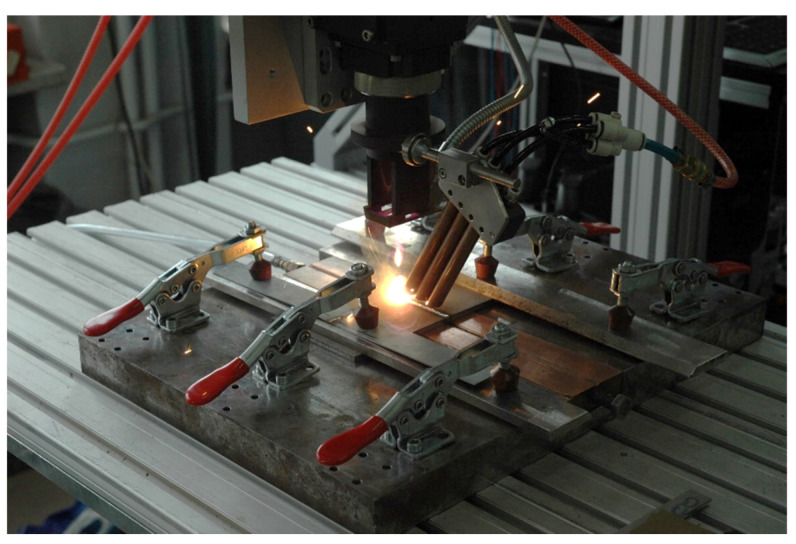
The autogenous laser welding of butt joints of Armox 500T steel samples.

The specimens to be welded were mounted in a stiffening clamping device with backing gas flow (argon of purity 99.999%) to protect the root side of the weld against ambient air. The argon flow from the root side was kept constant at 5.0 L/min. The top side of the welding zone was protected by argon flow via four cylindrical nozzles 8.0 mm in diameter each, and set at an angle of 45° to the sample surface. The flow of argon was kept at approx. 20.0 L/min. The experimental setup and a view of laser welding is shown in Figure 1.

When the laser welding tests were completed, first the visual inspections (VT) were performed according to PN-EN ISO 17637:2017-02 standard [25]. Next the metallographic examinations were carried out. The samples for metallographic examinations were cut perpendicularly to the weld axis away from the region of initiation and completion of welding.

The samples were mounted in thermosetting phenol resin with graphite filler Electro-WEM (Metalogis, Warsaw, Poland), and next the samples were wet ground via water papers with grit 120 to 2500 using an automatic grinding/polishing machine Struers Labopol-2 (Struers, Rodovre, Denmark). Next the cross-sections were polished with 1 µm diamond suspension Metkon Diapat-M (Metkon Instruments Inc., Bursa, Turkey). After polishing, the cross-sections were etched via Nital solution (3.0%).

Macrostructure analyses were carried out on the OLYMPUS SZX9 microscope (Olumpus Corporation, Tokyo, Japan), while the microstructure observations were performed with a NIKON Eclipse MA100 microscope (Nikon Corporation, Tokyo, Japan). The microstructure was also examined via scanning electron microscopy (SEM) (Carl Zeiss, Oberkochen, Germany), equipped with the energy dispersive spectrometer (EDS) (Oxford Instruments, Abingdon, UK). The phase composition was determined via X-ray diffraction (Panalitycal, Almelo, The Netherlands) with CuKα source of radiation andwith the scanning range of the diffraction angle 2θ from 0 to 140°. Chemical composition of the investigated steel was determined via a glow discharge spectrometer GDS850 (LECO, Geleen, The Netherlands).

The hardness distribution was measured on the cross-section of the test butt joints via the hardness tester WILSON WOLPERT 401 MVD (Wolpert Wilson Instruments, Aachen, Germany) at the load 5 N and the dwell time 10 s. The measurements were conducted in the middle of the plate thickness according to the PN-EN ISO 9015-2:2016-04 standard [26].

Mechanical properties of test joints were determined via the technological bending test (PN-EN ISO 5173:2023-06 [27], static tensile test (PN-EN ISO 148-1:2017-02 [28]), and Charpy V–notch test (PN-EN ISO 4136:2022-12 [29]).

The typical size of samples for impact tests according to the standard is 10 × 10 mm, but the standard allows samples with a smaller thickness, even up to 2.5 mm. In this case it is necessary to specify the thickness and total cross-section. The impact tests were carried out for at least 3 samples. The V–notch was cut in the weld from the face side. A load corresponding to impact energy of 150 J was used for the tests.

Samples for bending test were 20.0 mm wide and two samples were taken from each tested weld joint. The bending mandrel with a diameter of 35 mm was chosen according to the standard for a plate thickness of 3.0 mm and elongation of the steel A = 8%, Table 2.

The samples for the static tensile test were 25.0 mm wide in the gripping part, 12.0 mm wide and 70 mm long in the measuring part. The jaw travel speed was 0.008 mm/s.

## 3. Results and Discussion

### 3.1. Testing the Shape of Fusion Zone and the Quality of Bead-on-Plate Welds

**Figure 2:**
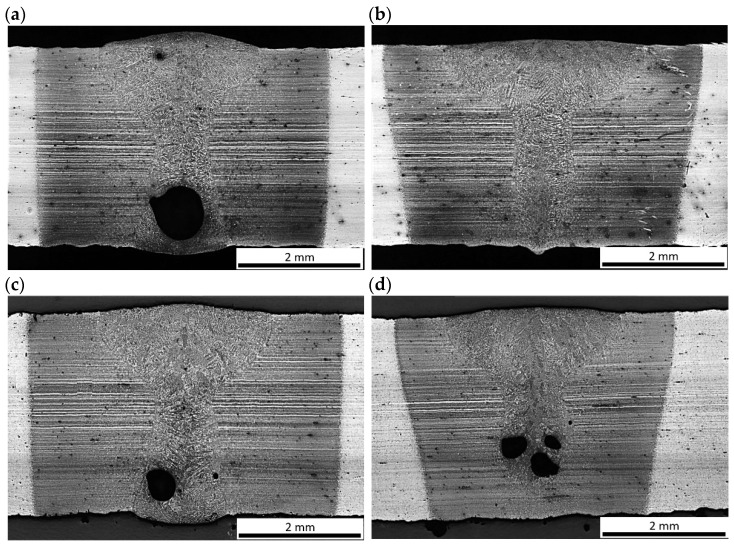
Influence of focal position of the laser beam on the penetration depth and shape of the fusion zone during bead-on-plate laser welding at laser output power 1.25 kW, welding speed 0.5 m/min (energy input 150 J/mm, Table 3); focal position: (**a**) B2, f: 0 mm, (**b**) B3, f: 1.5 mm, (**c**) B4, f: −1.5 mm, and (**d**) B5, f: −3.0 mm.

The preliminary tests were conducted for the simulation of an autogenous laser welding process of butt joints of 3.0 mm thick Armox 500T steel plates. The aim of this stage of the study was to establish the range of basic laser welding parameters for butt joints, providing full penetration and accepted quality of the welds. The influence of the laser beam focal position on the penetration depth and shape of the fusion zone during bead-on-plate laser welding at laser output power was 1.25 kW, with a welding speed of 0.5 m/min, and an energy input of 150 J/mm (Figure 2). As can be seen, the geometry of the welds produced with the laser beam focused on the top surface (Figure 2a), and with the beam focused 1.5 mm under the top surface (Figure 2c) was almost identical, Table 3. In this case, the width of the weld face was 2.4–2.5 mm, while the width of the weld root was 1.3 mm. Therefore, the location of the focal plane in the range from the top surface to the center of the plate thickness (focal position from 0 to −1.5 mm) did not affect significantly the shape of the fusion zone and penetration depth. The shape of the fusion zone of both welds was in X configuration (also called the hourglass shape), and the depth/width ratio of FZ 1.25 indicates that the welds were produced at keyhole mode welding. Further lowering the focal position led to lack of penetration, and at the same time widening of the weld face to 2.7 mm as can be seen in Figure 2d. Focusing the laser beam above the top surface of the plate was also unfavorable, as it led to a change in shape to a Y configuration (also called as mushroom shape) and a reduction in width of the root, Figure 2b. In this case, the width of the weld face increased to approx. 3.0 mm, while the width of the weld root was reduced to only 0.3 mm. Moreover, the clear tendency of porosity formation mainly in the lower region of the welds was found in the range of the investigated parameters of welding, regardless of whether there was a full penetration or not.

**Figure 3:**
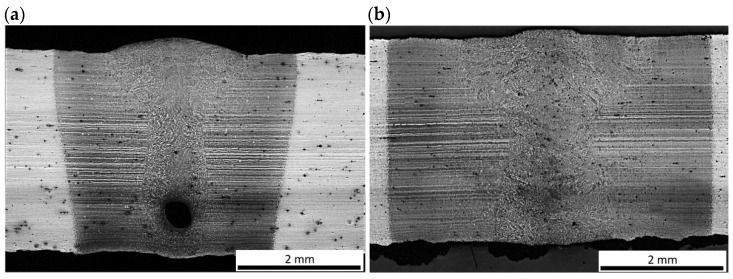
Influence of laser output power on the shape of fusion zone during bead-on-plate laser welding at welding speed 0.5 m/min and laser beam focused on the top surface (focal position, f 0 mm, Table 3); laser output power: (**a**) B1, 1.0 kW (energy input 120 J/min), (**b**) B6, 1.5 kW (energy input 180 J/min).

**Figure 4:**
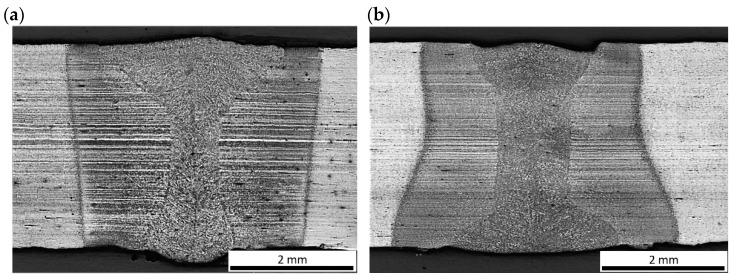
Comparison of the shape of fusion zone of welds produced during bead-on-plate laser welding at constant energy input of 90 J/mm, but different laser output power and welding speed (Table 3); (**a**) B7, laser output power 1.5 kW, welding speed 1.0 m/min; and (**b**) B8, laser output power 3.0 kW, welding speed 2.0 m/min.

The influence of laser output power increase by 50% on the penetration depth and shape of the fusion zone during bead-on-plate laser welding at welding speed 0.5 m/min is shown in Figure 3. Laser welding at the laser output power 1.0 kW and welding speed 0.5 m/min, simply calculated via the energy input (ignoring the heat transfer coefficient) 120 J/mm, resulted in a weld face width of 2.0 mm but also lack of penetration, Figure 3a. Increasing the laser output power to 1.5 kW provided full penetration of the steel plate and resulted in an increase in the width of the weld face to 2.6 mm. The width of the root was 1.8 mm in this case, Figure 3b. While it is obvious that the increase in laser power resulted in an increase in heat input of welding and therefore, led to increase in penetration and width of the fusion zone, it should be noted that the energy input is a simplified parameter and does not directly determine the shape of the weld, especially in the case of laser welding in keyhole mode. This phenomenon is illustrated in Figure 4 which shows different geometry of welds produced at the same energy input 90 J/mm. The weld shown in Figure 4a, produced at a laser output power 1.5 kW and a welding speed 1.0 m/min, exhibits a Y shape fusion zone, with a weld face width of 2.7 mm and a root width of 1.2 mm. While the weld shown in Figure 4b, produced at double the laser output power (3.0 kW) and a welding speed (2.0 m/min), exhibits an X shape fusion zone, with a weld face width of 1.8 mm and a root width of 2.5 mm. Due to the significant difference in welding speed, despite constant energy input, the mechanisms of material heating, melting, solidification, and heat transfer are different. Moreover, the power density of the laser beam, as a heat source, affects the absorptivity of laser radiation and thus the efficiency of heat transfer. Therefore, the simply calculated energy input (based on power and welding speed) is not a determinant of the real heat input, which depends also on the efficiency of heat transfer.

The results of bead-on-plate laser welding also showed that the Y shape of welds and low welding speed of 0.5 m/min favored porosity mainly in the lower area of the weld (root porosity), Figure 2, Figure 3 and Figure 4, and Table 3. This type of porosity is typical of laser welding in keyhole mode. Such porosity occurs usually within a certain range of welding parameters, and this phenomenon is related to the convection flow of liquid metal and partial evaporation. Many research results on the phenomenon of weld porosity during keyhole laser welding have been presented so far [18,21,22,23]. Some of the researchers found that the size and type of weld porosity (e.g., uniform, transitional, or root) is related mainly to the laser welding speed. In turn, other researchers pointed out that the porosity depends on the weld geometry (depth/width ratio). However, the geometry of the weld (fusion zone) during laser welding is related to the power density of the laser beam on the material (laser beam power relative to the surface area on which it affects, W/cm^2^), and thus spot size, focal plane position, and also heat input of laser welding (both welding speed and laser power). Under such conditions the unstable keyhole may easily collapse. Therefore, the collapsing molten metal from the top regions of the keyhole is thought to trap the gases and vapors in the solidifying weld leading finally to pore formation in the weld metal [22,23,24,30,31,32].

### 3.2. Microstructure and Hardness Distribution of Butt Joints

**Figure 5:**
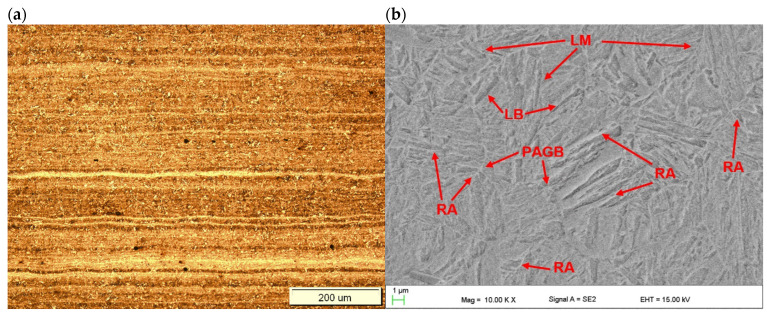
Microstructure of base metal of 3.0 mm thick Armox 500T steel plate (Table 1 and Table 2); (**a**) optical micrograph, (**b**) SEM micrograph; LM—lath martensite, PAGB—prior austenite grain boundary, LB—lath boundaries, RA—traces of retained austenite.

**Figure 6:**
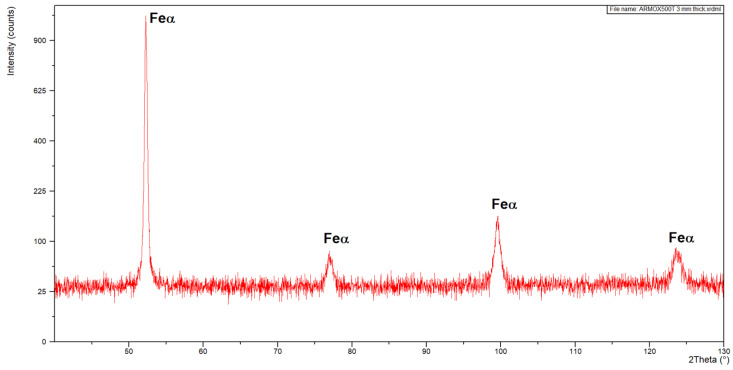
XRD pattern of the base metal Armox 500T steel plate 3.0 mm thick.

The microstructure of the base metal of 3.0 mm thick Armox 500T steel plate is shown in Figure 5. As can be seen in Figure 5a, the base metal is characterized by very fine grains with clear segregation shearing lines, expanding along the plate rolling direction, as a result of inhomogeneous plastic deformation, in subsequent rolling passes. Moreover, the microphotograph in Figure 5a may suggest the presence of dispersed fine precipitations. In turn, based on the chemical composition, it can be suspected that they were carbides. However, the results of the XRD analysis of the base metal of Armox 500T steel at delivery conditions revealed peaks only from Feα’ (martensite), see Figure 6. However, it should be noted that the detection level of the applied XRD method was about 3% for the specific γ phase. Therefore, it did not allow for the precise identification of the fine precipitations or phases at a share lower than 3%.

Moreover, detailed observation of the microstructure at high magnification on SEM micrographs indicated that the base metal consisted mainly of tempered lath martensite. Some traces of morphology typical for retained austenite can be also observed, as shown in Figure 5b [1,3,33,34]. Planimetric analysis of the morphology typical of retained austenite indicated its share of 1.5–2%. The typical microstructure of Armox 500T steel consists of martensite and a few percent of retained austenite, as provided by the manufacturer (SSAB), and proved by many studies [1,3,4,11].

However, it should be noted that the microstructure of the armor steel Armox 500T is highly dependent on the heat treatment conditions. The microstructure changes with decreasing cooling rate during hardening, from martensitic to martensitic-bainitic, bainitic, bainitic-ferritic-pearlitic, to pearlitic-ferritic [3,4,5].

The fine-grained tempered lath martensite microstructure with a small amount of retained austenite is most preferable from the point of view of ballistic performance [1,4,6]. Hard tempered martensite with fine morphology and with some fraction of stable retained austenite is able to absorb excess of strain energy through plastically induced transformation into bcc-martensite. The most preferable amount of retained austenite in the case of armor steel plates should be in the range of 3–5%, and additionally it should be stable. Heat treatment, which leads to the precipitation of carbides and causes a significant reduction in ballistic resistance which was described by El-Fawakhry et al. [3].

**Figure 7:**
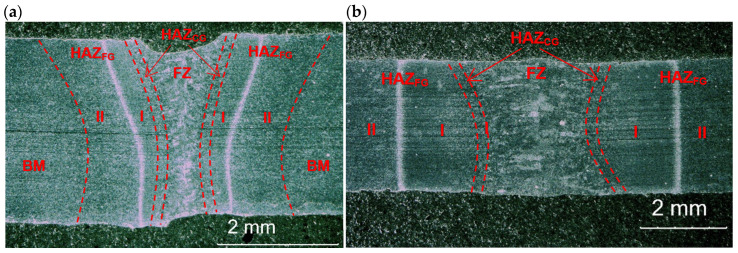
Cross-sections of the 3.0 mm thick butt joints of Armox 500T steel produced via autogenous laser welding (Table 4); (**a**) at lowest energy input of 70 J/mm (3.0 kW, 2.5 m/min), (**b**) at highest energy input of 180 J/mm (1.5 kW, 0.5 m/min); BM: Base Metal, FZ: Fusion Zone, HAZ_FG_: Fine-Grained Heat-Affected Zone, HAZ_FG_: Coarse-Grained Heat-Affected Zone.

Based on the initial tests of bead-on-plate laser welding, the extreme parameters with the highest and the lowest energy input, providing full penetration and required weld quality with limited tendency to porosity, were chosen for autogenous butt joint laser welding. The cross-sections of representative butt joints are presented in Figure 7. The butt joint produced at the lowest energy input of 70 J/mm shows just a slight undercut of the weld face (Figure 7a).

However, such weld face undercuts of not more than 10% of the plate thickness are typical and acceptable in the case of autogenous laser welding. In turn, the butt joint produced at the highest energy input 180 J/mm showed small single pores in the middle region of the weld, and with a diameter of not more than 125 µm (Figure 7b). The shape and the width of the weld and heat-affected zone are obviously related to the energy input. It is worth noting that the shape (configuration of fusion zone) and dimensions of the butt joints differed to the bead-on-plate welds. This phenomenon is related to the different conditions of melting and solidification of I-joint type during autogenous welding (without filler).

**Figure 8:**
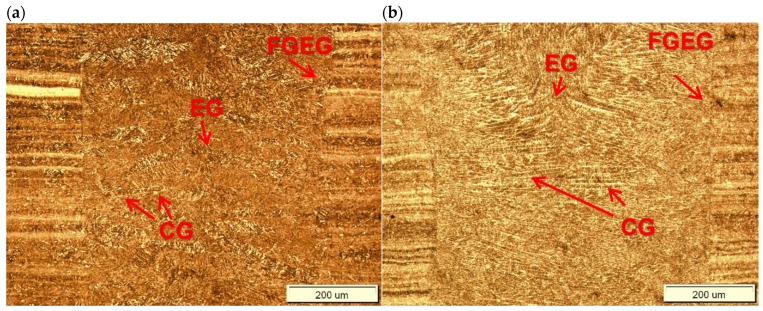
Optical micrographs of the middle region of fusion zone of 3.0 mm thick butt joints of Armox 500T steel produced via autogenous laser welding (Table 4); (**a**) at lowest energy input of 70 J/mm (3.0 kW, 2.5 m/min), (**b**) at highest energy input of 180 J/mm (1.5 kW, 0.5 m/min); CG: Columnar Grains, EG: Equiaxed Grains, FGEG: Fine-Grained Equiaxed Grains.

**Figure 9:**
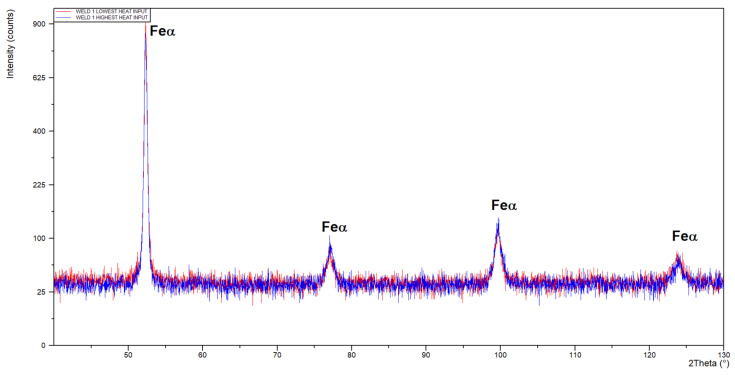
XRD pattern of butt joints of Armox 500T 3.0 mm thick steel produced via autogenous laser welding.

**Figure 10:**
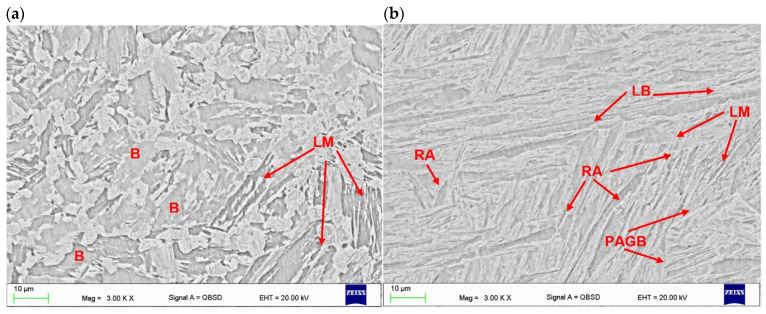
SEM micrographs of butt joints produced the at lowest energy input of 70 J/mm (Table 4); (**a**) HAZ adjacent to the fusion line, (**b**) fusion zone in the center region of weld; LM—lath martensite, PAGB—prior austenite grain boundary, LB—lath boundaries, B—bainite, RA—traces of retained austenite.

**Figure 11:**
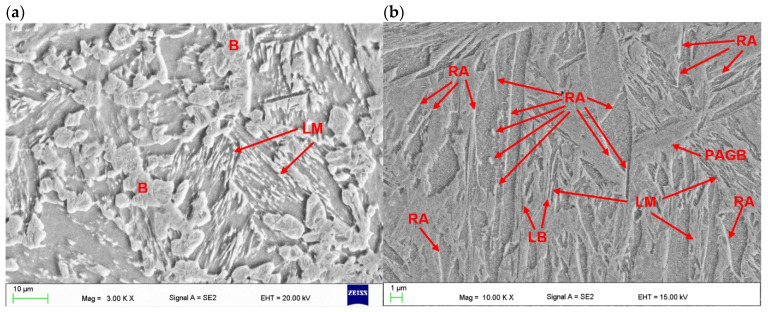
SEM micrographs of butt joints produced the at highest energy input of 180 J/mm (Table 4); (**a**) HAZ adjacent to the fusion line, (**b**) fusion zone in the center region of weld; LM—lath martensite, PAGB—prior austenite grain boundary, LB—lath boundaries, B—bainite, RA—traces of retained austenite.

The optical micrographs of the central region of fusion zone, along with the fusion line and HAZ of the butt joints are shown in Figure 8. As can be seen, the fusion line was sharp and clear. Therefore, the coarse-grained region of HAZ, adjacent to the fusion line, was very narrow in the case of both butt joints. In this region, due to high temperature, a slight grain growth occurred if compared to the microstructure of the base metal. Due to recrystallization the austenite grains transformed into martensite with possible content of bainite and retained austenite. The XRD spectra of weld metal taken from both joints produced at different parameters were very similar with peaks only from Feα’ (martensite), as can be seen in Figure 9. However, traces of the morphology typical for retained austenite in weld metal can be also observed in SEM micrographs, as shown in Figure 10b and Figure 11b.

In turn, the fine-grained region and partially transformed region of HAZ was directly dependent on the energy input, as can be seen in Figure 7 and Figure 8.

It should be noted that the HAZ did not end at the clearly visible “white line”, but extended much further, as marked with red dashed lines in Figure 7a,b. The actual width of the HAZ was identified during measurements of microhardness distribution on the cross-section of the test joints. Additionally, the fine-grained region of HAZ was divided into two areas marked as I and II, due to the different distribution of microhardness. As can be see, the total width of the HAZ was twice to three times larger than the width of the fusion zone.

The SEM micrographs taken from the “white line” zone of fine-grained HAZ of butt joints produced at different parameters is shown in Figure 10a and Figure 11a.

As can be seen, the microstructure of this zone differed from the base metal and fusion zone, and the share of martensite was much smaller and its morphology was different (small grained martensite), see Figure 10a and Figure 11a. Other researchers also point to the existence of a soft zone with soft patches of white ferrite phase, placed between the fine-grained HAZ and base metal [6,18]. However, accurate microhardness measurements on the cross-section of the joints allowed for the precision location of areas with significant changes in hardness. The area of lowest hardness, where the hardness drops off rapidly, is the clearly visible “white line” in the fine-grained HAZ. In turn, between the fusion line and the “white line”, the hardness even increases. SEM images of the microstructure in the “white line” zone located in the fine-grained HAZ of both tested test joints indicated the dominant share of bainite in relation to martensite, see Figure 10a and Figure 11a.

The optical micrographs of the joints in the central region of welds exhibited typical primary structure with columnar grains growing from partially melted grains at the fusion line and equiaxed grains in the central region of the fusion zone (Figure 8). The structure of the fusion zone and the share of columnar and equiaxed grains obviously depended on the thermal conditions and heat input during laser welding.

The primary structure of the weld was formed during its solidification. The weld produced at the higher heat input exhibited a thin, fine-grained zone of equiaxed grains in the vicinity of the fusion line, which grew on partially melted grains of the base metal. The cooling rate during solidification in this region was the highest and the molten metal was highly supercooled. The width of this zone was approx. 40–50 µm, as can be seen in Figure 8b. When this zone was formed, it reduced the cooling rate, which reduced supercooling and the rate of nucleation as well. Therefore, this led to the formation of a zone of large columnar grains with a privileged crystallographic orientation, and the direction of their rapid growth coincided with the direction of heat dissipation. The solidification of the central zone of the weld took place with the lowest supercooling and without a clear direction of heat dissipation. Randomly oriented nuclei passed through the dendritic crystallization phase and grew to size depending on the thermal conditions of the welding process. As can be seen in Figure 8b, the columnar grain size is approx. 250–300 µm, which almost converged in the middle of the weld, due to which the area of equiaxed grains in its region is narrow.

**Figure 12:**
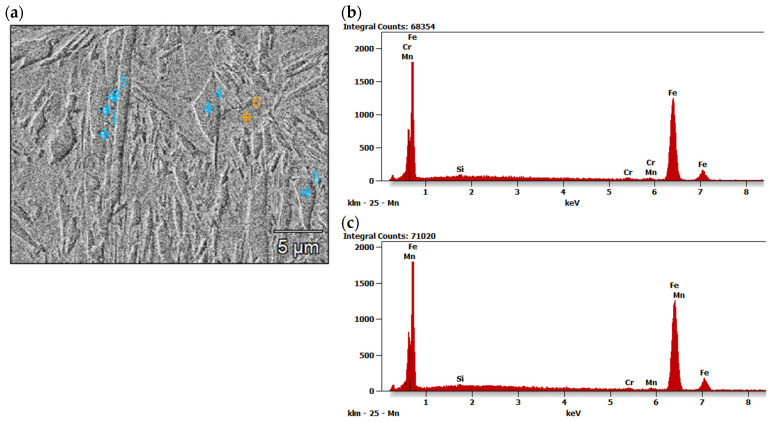
SEM micrographs of fusion zone of butt joint produced the at highest energy input of 180 J/mm (**a**), representative EDS spectra taken from point 1 (**b**) and point 4 (**c**).

On the other hand, the structure of the weld made with lower energy input was different, mainly due to the lower proportion of columnar grains, as can be seen in Figure 8a. This was related to the higher rate of cooling, crystallization, and thus the difference in the primary structure. It should be also noted that the structure on the cross-section of the weld was not homogeneous. The results of EDS analysis of some constituents of the weld metal of the joint produced at higher energy input (180 J/mm) are presented in Figure 12, Table 5. However, the higher cooling rate affected the extent and intensity of the martensitic transformation in the solid state, and thus the final microstructure of the weld. Moreover, the size of martensite laths in the grains also depended on the heat input. Higher heat input led to coarsening of the laths of martensite, as can be seen in Figure 10b and Figure 11b.

**Figure 13:**
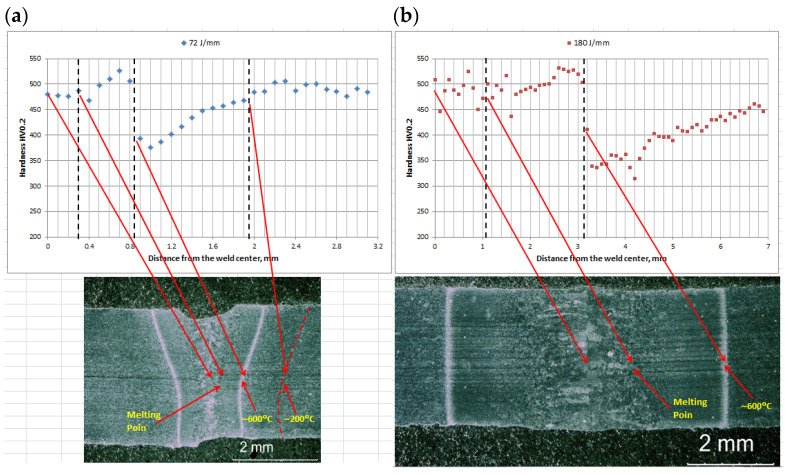
Microhardness distribution on the cross-section of 3.0 mm thick butt joints of Armox 500T steel produced via autogenous laser welding (Table 4); (**a**) at lowest energy input of 70 J/mm (3.0 kW, 2.5 m/min), (**b**) at highest energy input of 180 J/mm (1.5 kW, 0.5 m/min).

Microhardness measurements conducted on cross-sections of the butt joints showed that the base metal of Armox 500T steel was characterized by the microhardness ranging in 470–510 HV0.2 (Figure 13).

The microhardness distribution across the joint welded at lower energy input of 70 J/mm was very uniform with low scatter. The microhardness of the fusion zone was slightly lower compared to the BM from 477 to 480 HV0.2 (Figure 13). The highest values up to 526 HV0.2 were determined in the first region “I” of HAZ, see Figure 7a and Figure 13a. As can be seen in Figure 13a, the rapid drop of the microhardness to375–390 HV0.2 occurred exactly at the sharp “white line”. Then the microhardness increased gradually in the second region “II” of HAZ up to the microhardness of the base metal. A similar microhardness distribution occurred in the case of the joint welded at higher energy input of 180 J/mm. However, the width of the characteristic zones was larger, the microhardness values were different and the scatter of results was greater. It is worth noting that the microhardness was measured along three parallel lines, but the error bars were omitted so as not to reduce the clarity of the graphs, especially for the joint welded with higher energy input. As can be seen, the microhardness in the fusion zone varied in range from 446 HV0.2 to 525 HV0.2 (Figure 13b). There was also significant scatter of result in the coarse-grained HAZ adjacent to the fusion line. In this region the microhardness varied from 437 HV0.2 to 517 HV0.2 (Figure 13b). Similar to the previous joint, the maximum value of microhardness 531 HV0.2 was determined in the first region “I” of HAZ. The drop in microhardness at the “white line” zone was even greater and reached 315–345 HV0.2 (Figure 13b). Although, the decrease was significant, the microhardness level was typical of bainitic structure rather than ferritic structure.

The hardness distribution was obviously related to the change in microstructure caused by the thermal cycle, i.e., the temperature range and cooling rate. Determining the temperature distribution in the HAZ under laser welding conditions is not easy due to the high temperature gradient and high traveling speed of the heat source. However, it can be assumed that at the fusion line the temperature reaches the melting point, as indicated in Figure 13. On the other hand, the influence of heat treatment of Armox 500T steel on hardness, tensile strength, and impact strength is well studied and there are many articles in this area. Hyeon-Seok et al. [35] found that there is no significant decrease in hardness at the tempering temperature of 150–200 °C. In turn, at the tempering temperature of 600 °C, the hardness decreased to approximately 320 HV [35].

The sudden drop in microhardness in the HAZ can be explained after estimating the temperature distribution in this area. If it is assumed that the hardness in the HAZ is proportional to the tempering temperature of Armox 500T steel, then characteristic temperature can be assigned to the boundary lines, as shown in Figure 13. An increase in the tempering temperature of Armox 500T steel up to 600 °C generally leads to coarsening of the martensite laths, carbon diffusion and a decrease in the dislocation density in martensite, as well as carbide precipitation. In turn, in the HAZ area, where the temperature is between 700 °C up to the fusion line, recrystallization of the primary structure takes place.

In the earlier work Lisiecki A. [15] proposed a methodology for determining the cooling time in the temperature range of 800 and 500 °C (t_8/5_), i.e., in the HAZ recrystallization zone under autogenous laser welding conditions. Since the detailed methodology for determining the cooling time t_8/5_ is presented in this reference [15], it was omitted in the current work. The cooling times determined according to this methodology for the butt joints of Armox 500T were 0.45 s for the heat input of 70 J/mm (BJ2) and 2.98 s for the heat input of 180 J/mm (BJ1), respectively.

### 3.3. Mechanical Properties and Fracture Mode of Butt Joints

**Figure 14:**
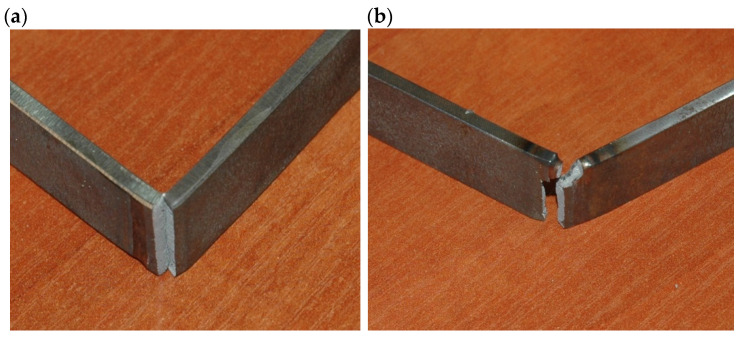
A view of the samples after technological bend test (weld root bending) of 3.0 mm thick butt joints of Armox 500T steel produced via autogenous laser welding (Table 4 and Table 6); (**a**) at lowest energy input of 70 J/mm (3.0 kW, 2.5 m/min), (**b**) at highest energy input of 180 J/mm (1.5 kW, 0.5 m/min).

The results of technological bending test of butt joints of 3.0 mm thick Armox 500T steel autogenously laser welded revealed low plasticity of the welds and HAZ of the joints. While the base metal of Armox 500T steel should withstand a full bending angle of 180°, the butt joint’s failure occurred at a bending angle of just 25–35°, Figure 14, Table 6.

**Figure 15:**
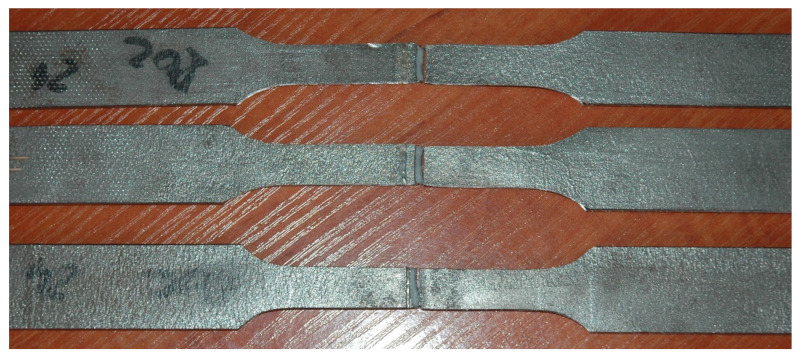
A view of broken samples after the static tensile tests of butt joint of Armox 500T steel produced via autogenous laser welding (Table 4) at lowest energy input of 70 J/mm.

The results of static tensile test of butt joints of 3.0 mm thick Armox 500T steel autogenously laser welded has shown satisfactory ultimate tensile strength, especially in the case of the joint welded at the highest energy input, although the rupture occurred at the HAZ location with the lowest hardness. The mean value of tensile strength for samples of the joint welded at 180 J/mm was over 1400 MPa (Figure 15 and Table 7).

On the other hand, the joint welded at the lowest energy input of 70 J/mm showed lower tensile strength, which was related to the crack location at the fusion zone boundary (Figure 15 and Table 7). However, in this case, the zone between the fusion line and the region of the lowest microhardness was very narrow, approximately 0.5 mm, so it was not possible to clearly identify the place where the crack was initiated. Additionally, a decrease in hardness was also identified near the fusion line, which may indicate a decrease in strength in this area, Figure 13a.

The hardness drop and weakening of the material in HAZ was related with coarsening the microstructure due to thermal cycle, especially in the region of coarse-grained HAZ but also as a result of tempering and the related structure transformation resulted in the formation of bainitic microstructure in a certain zone; this can be seen as a white line in the HAZ. Moreover, in the case of the investigated steel the thermal cycle in HAZ may lead to the transformation of the retained austenite into bainite, which is less ductile, and also to precipitation of complex carbides such M_23_C_6_ and M_7_C_3_ [24,30,31,32,34,36,37].

The results of Charpy V–notch test of the butt joints 3.0 mm thick of Armox 500T steel produced via autogenous laser welding at different energy inputs, tested at room temperature, and also at reduced temperature are summarized in Table 7. As can be seen, the impact toughness of the welded joints tested at 20 °C (room temperature) was at the level required for the base metal of Armox 500T steel at −40 °C, Table 2 and Table 7. In turn, the impact toughness of the welded joints tested at −20 °C is approximately 25–30% lower than values determined at room temperature.

**Figure 16:**
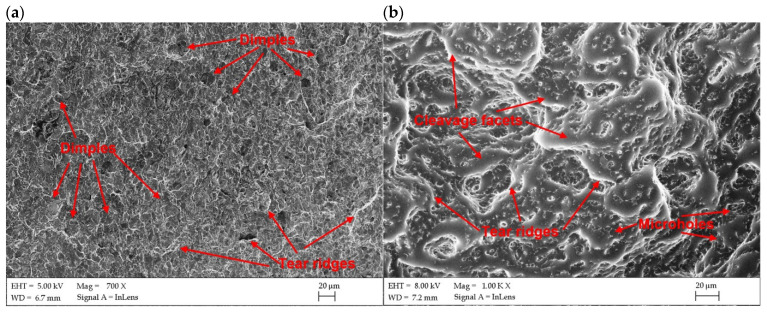
SEM images of fracture surfaces of Charpy impact samples of butt joints produced the at lowest energy input of 70 J/mm (Table 4); (**a**) tested at 20 °C, (**b**) tested at −20 °C.

**Figure 17:**
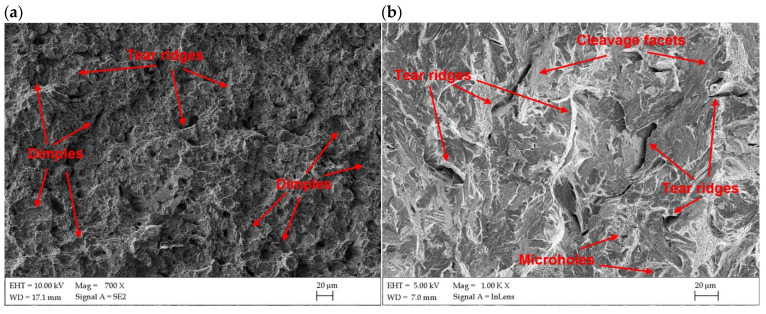
SEM images of fracture surfaces of Charpy impact samples of butt joints produced the at lowest energy input of 180 J/mm (Table 4); (**a**) tested at 20 °C, (**b**) tested at −20 °C.

The fracture surface of the Charpy impact samples were examined under SEM microscope to determine the mode of fracture of butt joints produced at different heat input and different temperature. As can be seen in Figure 16b and Figure 17b, the fracture surface of samples tested at −20 °C showed mainly cleavage fracture mode for both joints. The cleavage facets and very limited amount of dimples, as well as microvoids and holes can be observed on the fracture surface in the case of joint produced at the lowest energy input of 70 J/mm (Figure 16b). In turn, the fracture surface of the joint produced at the higher energy input of 180 J/mm shows rather quasi-cleavage facets with clear shearing areas and tear ridges (Figure 17b). This also corresponded with the higher impact energy absorbed during the tests by this joint (9.5 J), compared to the joint produced at lower energy input (8.6 J), as seen in Table 8.

In turn, the samples tested at 20 °C (room temperature) indicated a mixed mode of fracture with dimples that can be seen on the surfaces. Such fracture surface topography indicated that the progress of damage partially followed a void growth and subsequent coalescence (Figure 16a,b). The fracture surfaces in Figure 16a and Figure 17a showed a large density of the fine dimples of different sizes. Some microvoids and holes can be also identified on fracture surfaces of both tested joints. However, more ductile dimples may be observed on the fracture surface of the joint produced at higher energy input. The results of impact test also showed higher impact energy absorbed by the joint (13.3 J), compared to the joint produced at a lower energy input (11.4 J).

In general, the drop of impact toughness of weld metal was mainly related to the grain size coursing and grain orientation [24,30,33]. Considering that higher energy input of laser welding leads to grain growth, it can be concluded that the grains orientation had a greater influence on the impact characteristics of the tested butt joints produced within the specific parameters and conditions of welding.

## 4. Conclusions

Based on the results of the study on autogenous laser welding of 3.0 mm thick Armox 500T plates, the following conclusions were drawn:

- Low welding speed of 0.5 m/min and Y-type shape of fusion zone favor porosity in the bottom of the weld (root porosity), which is a common problem during keyhole laser welding.
- Welding at higher speed provided a columnar (I configuration) or hourglass (X configuration) shape of the fusion zone, and eliminated the risk of porosity. Therefore, it was possible to ensure satisfactory quality of butt joints, characterized by a proper geometry, and no internal defects.
- Microstructure of the fusion zone and HAZ was related directly to the energy input of laser welding, thus thermal conditions of solidification and cooling rates. The dominant phase constituent in the fusion zone and HAZ was martensite. The fusion zone consisted of columnar and equiaxed grains with the size of martensite dependent on the energy input of laser welding process.
- The butt joints were characterized by significantly lower microhardness in HAZ due to transformation of microstructure into bainitic in the specific zone. Therefore, the static tensile strength of the joints was lower that the base metal. Impact tests conducted at reduced temperature (−20 °C) showed a reduction of the impact toughness by approx. 25–30%, if compared to the values achieved at room temperature. The fracture surface of samples tested at −20 °C exhibited clearly a cleavage fracture mode, while the samples tested at 20 °C exhibited a mixed mode of fracture with some dimples.
- Narrow weld and HAZ may be beneficial from the point of view of the ballistic performance of the butt joint of armor plates.
- It is planned to continue research in the field of laser welding greater thicknesses of armor plates.

## Figures and Tables

**Table 1:** Chemical composition of Armox 500T steel (wt%).

Accor. to	C	Mn	Si	P	S	Cr	Ni	Mo	B
SSAB	0.32	1.2	0.4	0.01	0.003	1.0	1.8	0.7	0.005
Tested	0.28	1.1	0.2	-	-	0.88	1.4	0.6	-

Remarks: SSAB: results provided by the manufacturer, tested: GDS analysis of 3.0 mm thick Armox 500T plate.

**Table 2:** Mechanical properties of Armox 500T steel (wt%).

Accor. to	Hardness, HBW	Minimum Yield Strength Rp_0.2_, MPa	Tensile Strength Rm, MPa	Total Elongation, A_5_, %	Total Elongation, A_50_, %	Notch Impact Energy at −40 °C, KV, J
SSAB	480–540	1250	1450–1750	8	10	32
Tested	-	1298	1553	8.2	11	22

Remarks: SSAB: results provided by the manufacturer, tested: results determined during mechanical testing of 3.0 mm thick Armox 500T plate.

**Table 3:** Parameters of bead-on-plate laser welding of the 3.0 mm thick plates of Armox 500T steel.

Weld Bead No.	Output Laser Power kW	Welding Speed m/min	Focal Position * mm	Energy Input ** J/mm	Width of the Weld Face mm	Width of the Weld Root/Penetration Depth mm	Fusion Zone Shape	Remarks
B1	1.0	0.5	0	120	2.0	-/2.7	Y	L, P
B2	1.25	0.5	0	150	2.4	1.3	X	L, P
B3	1.25	0.5	1.5	150	3.0	0.3	X	F
B4	1.25	0.5	−1.5	150	2.5	1.3	Y	F, P
B5	1.25	0.5	−3.0	150	2.7	-/2.5	Y	L, P
B6	1.5	0.5	0	180	2.6	1.8	X	F
B7	1.5	1.0	0	90	2.7	1.2	Y	F
B8	3.0	2.0	0	90	1.8	2.5	X	F
B9	3.0	2.5	0	70	1.4	2.3	X	F,H

Remarks: * position relative to the top surface of plate (negative value means focusing below the surface); ** energy input is simply calculated by considering laser output power and welding speed only, while the heat input also takes into account the heat transfer coefficient; Quality assessment: L—lack of fusion, F—full penetration, H—hollow face, P—porosity.

**Table 4:** Parameters of autogenous butt joint laser welding of the 3.0 mm thick plates of Armox 500T steel.

Weld Bead No.	Output Laser Power kW	Welding Speed m/min	Focal Position * mm	Energy Input J/mm	Width of the Weld Face mm	Width of the Weld Root	Fusion Zone Shape	Remarks
BJ1	1.5	0.5	0	180	2.6	2.8	X	FP
BJ2	3.0	2.5	0	70	2.5	1.3	Y	FP,U

Remarks: * position relative to the top surface of plate; Quality assessment: LF—lack of fusion, FP—full penetration, U—undercut of wed face.

**Table 5:** Quantitative results of EDS analysis in different points in fusion zone of butt joint produced the at highest energy input (Figure 12a).

Tested Region	Si		Cr		Mn		Fe	
	Wt.%	At.%	Wt.%	At.%	Wt.%	At.%	Wt.%	At.%
Point 1	0.4	0.8	0.9	1.0	1.4	1.4	97.3	96.8
Point 2	0.4	0.7	1.1	1.1	1.1	1.2	97.4	97.0
Point 3	0.3	0.7	1.0	1.0	1.1	1.1	97.6	97.2
Point 4	0.3	0.6	0.7	0.7	0.9	1.0	98.1	97.7
Point 5	0.4	0.9	0.8	0.9	1.8	1.9	96.9	96.4
Point 6	0.5	1.1	0.9	1.0	1.3	1.3	97.2	96.6

**Table 6:** Results of the technological bending test of 3.0 mm thick butt joint of Armox 500T steel produced via autogenous laser welding (Table 4), according to the PN-EN ISO 5173:2023-06 standard, Figure 14.

Joint/Sample Type	Test Type	Bending Angle, °	Remarks/Location of Failure
JA (70 J/mm)	Face bend	35	Longitudinal and transverse fracture along the entire width/HAZ/FZ
	Root bend	32	Longitudinal fracture along the entire width/HAZ
JB (180 J/mm)	Face bend	25	Longitudinal fracture along the entire width/HAZ
	Root bend	28	Longitudinal fracture along the entire width/HAZ

Remarks: HAZ—heat-affected zone, FZ—fusion zone.

**Table 7:** Results of static tensile test of 3.0 mm thick butt joint of Armox 500T steel produced via autogenous laser welding (Table 4), according to the PN-EN ISO 148-1:2017-02 standard, Figure 15.

Joint Type	Sample No.	Tensile Strength Rm, MPa	Location of Failure
JA (70 J/mm)	JA1	1373.09	FZ/HAZ
	JA2	1369.14	FZ/HAZ
JB (180 J/mm)	JB1	1402.39	HAZ (weakest zone)
	JB2	1401.29	HAZ (weakest zone)

Remarks: HAZ—heat-affected zone, FZ—fusion zone, BM—base metal.

**Table 8:** Results of Charpy V–notch test of 3.0 mm thick butt joint of Armox 500T steel produced via autogenous laser welding (Table 4), according to the PN-EN ISO 4136:2022-12 standard, see Figure 16 and Figure 17.

Joint Type	Temperature of Charpy V–Notch Test	Mean Value of Absorbed Impact Energy, J	Mean Value of Impact Toughness, J/cm^2^
JA (70 J/mm)	20 °C	11.4	34.2
	−20 °C	8.6	25.8
JB (180 J/mm)	20 °C	13.3	39.9
	−20 °C	9.5	28.5

## Data Availability

The original contributions presented in the study are included in the article, further inquiries can be directed to the corresponding author.
